# The influence of age, gender and education on the performance of healthy individuals on a battery for assessing limb apraxia

**DOI:** 10.1590/S1980-5764-2016DN1003010

**Published:** 2016

**Authors:** Joana Mantovani-Nagaoka, Karin Zazo Ortiz

**Affiliations:** 1 Speech Therapist, Master in Sciences, Department of Human Communication Sciences, Universidade Federal de São Paulo, SP, Brazil.; 2 Specialist, Master, and PhD in Human Communication Disorders. Postdoctoral fellow in Neuroscience. Associate Professor, Department of Human Communication Sciences, Universidade Federal de São Paulo, SP, Brazil.

**Keywords:** apraxia, ideomotor apraxia, educational status, age

## Abstract

**Introduction:**

Apraxia is defined as a disorder of learned skilled movements, in the absence of elementary motor or sensory deficits and general cognitive impairment, such as inattention to commands, object-recognition deficits or poor oral comprehension. Limb apraxia has long been a challenge for clinical assessment and understanding and covers a wide spectrum of disorders, all involving motor cognition and the inability to perform previously learned actions. Demographic variables such as gender, age, and education can influence the performance of individuals on different neuropsychological tests.

**Objective:**

The present study aimed to evaluate the performance of healthy subjects on a limb apraxia battery and to determine the influence of gender, age, and education on the praxis skills assessed.

**Methods:**

Forty-four subjects underwent a limb apraxia battery, which was composed of numerous subtests for assessing both the semantic aspects of gestural production as well as motor performance itself. The tasks encompassed lexical-semantic aspects related to gestural production and motor activity in response to verbal commands and imitation.

**Results:**

We observed no gender effects on any of the subtests. Only the subtest involving visual recognition of transitive gestures showed a correlation between performance and age. However, we observed that education level influenced subject performance for all sub tests involving motor actions, and for most of these, moderate correlations were observed between education level and performance of the praxis tasks.

**Conclusion:**

We conclude that the education level of participants can have an important influence on the outcome of limb apraxia tests.

## RESUMO

**Introdução:**

A apraxia é definida como sendo um distúrbio na realização de gestos ou atos motores aprendidos. Há poucos estudos sobre a avaliação da praxia de membros incluindo o controle de variáveis sócio-demográficas.

**Objetivo:**

O presente estudo teve por objetivo avaliar o desempenho de indivíduos saudáveis em uma bateria de praxia de membros, analisando-se a influência das variáveis sexo, idade e escolaridade nas habilidades práxicas avaliadas.

**Métodos:**

Quarenta e quatro indivíduos foram submetidos à aplicação de uma bateria de praxia de membros, composta de subtestes que avaliaram tanto aspectos semânticos relacionados à produção gestual, quanto a produção motora propriamente dita.

**Resultados:**

Não houve influência do sexo em nenhum dos subtestes. Apenas o subteste que envolvia o reconhecimento visual da correta realização de gestos transitivos, mostrou-se sensível à variação da idade. Já a escolaridade influenciou o desempenho dos participantes em todos os testes que envolviam a realização de atos motores, sendo que para a maior parte deles foram encontradas correlações moderadas entre a escolaridade e o desempenho nas tarefas práxicas.

**Conclusão:**

Variáveis sócio demográficas, principalmente a escolaridade, podem interferir no desempenho de indivíduos em testes que avaliam a praxia de membros e devem ser consideradas na avaliação clínica.

## INTRODUCTION

Apraxia is a well-known syndrome characterized by the patient's inability to perform routine gestures.^1^ It covers a wide spectrum of disorders, all involving motor cognition and the inability to perform actions that have been previously learned.^1^

Limb apraxia, a disorder of higher order motor control, has been a challenge for clinical assessment and understanding.^2^ The deficits originally described in limb apraxia^3^ have been classified by the nature of the errors made by patients leading to namely, ideational and ideomotor apraxia.^4^

Demographic variables such as gender, age, and education can influence the performance of individuals on different neuropsychological tests.^5^ ^-^ ^8^ Considering this, few studies have been carried out to specifically examine the influence of demographic variables on praxis skills, although there is recent research related to limb apraxia assessment.^9^ ^,^ ^10^ Therefore, the aim of this study was to evaluate the performance of individuals without neurological injury on a limb apraxia battery and to determine the influence of gender, age, and education on the assessed praxis skills.

## METHODS

The study was approved by the local Research Ethics Committee (Protocol Number 0170/05). After receiving full information about the study, written informed consent was obtained from all enrolled subjects. This study included 44 healthy subjects with no documented neurological deficits, no history of psychiatric treatment or psychotropic drug use, and no cognitive, behavioral and/or speech/language disorders, as determined by a brief neuropsychological battery.

Participants underwent a limb apraxia battery to evaluate all subcomponents of praxis processing, according to cognitive models.^1^ ^,^ ^11^ ^,^ ^12^ The battery comprised the following subtests:

### 1) Lexical-semantic aspects related to gestural production

1.1. Oral Comprehension of Actions and Objects: out of four photographs shown on a card, the participant must identify the picture corresponding to the action (10 items) or object (10 items) named by the evaluator. One point was given for each action and object correctly identified.

1.2. Naming Actions and Objects: the participant must name the action (10 items) or object (10 items) shown by the evaluator. One point was given for each action and object correctly named.

1.3. Recognition of Object Function: out of four photographs of objects displayed on a card, the participant must indicate the picture corresponding to the function described by the evaluator. One point was given for each object corresponding to the function description correctly identified.

1.4. Definition of Object Function: the participant must describe the function of 10 items shown to them. One point was given for each object function correctly described.

1.5. Comprehension of Transitive Gestures (i.e., gestures that involve the use of objects): Ten cards, each containing four photographs, are shown to the patient. In each figure, the same person uses the same object; however, in only one of these photos is the object being used correctly in terms of handling and spatial orientation. The participant must recognize and indicate the photo in which the object is being used correctly. One point was given for each correct use of the object indicated.

### 2) Motor activity in response to verbal commands and imitation^13^

2.1a. Ideomotor apraxia: the participant must demonstrate the use of a given object using gestures. For 3 items, the participant is allowed to hold the object in question in his/her hands, whereas for another 3 items, the evaluator simply states the object and the participant is asked to imagine the object in his/her hands and demonstrate (i.e., pantomime) its use. One point was given for each correct gesture corresponding to the object use.

2.1b. Static imitation of meaningless gestures: the participant is asked to imitate the evaluator's hand in three different positions. One point was given for each part of each one of the three positions performed correctly. 

2.1c. Dynamic imitation of meaningless gestures: the participant is instructed to accurately imitate the hand movements performed by the evaluator. One point was given for each part of each one of the three movements performed correctly. 

2.2. Ideational apraxia: performance of complex gestures is evaluated using 3 objects that are present and 3 objects that are absent (i.e., imagined).

One point was given for each part of each one of the six actions performed correctly.

2.3. Symbolic gestures: the meaning of three symbolic gestures is assessed: waving hands to say good bye, making a hand gesture to hail a taxi, and gesturing to make someone comes closer. One point was given for each gesture performed correctly.

Data collection was carried out on an individual basis by the same person.

All mistakes made were recorded.

Following completion of the battery, a Mann-Whitney test was used to compare results between genders. The age variable was analyzed as a continuous variable and Pearson's correlation was used to analyze the correlation between age and test performance. Education was analyzed as a categorical variable in terms of level of education and the Kruskal-Wallis test was used to analyze these data. Probability (p) levels less than 0.05 were considered to be statistically significant.

## RESULTS

Of the 44 participants in this study, 54.5% were women. The average age was 54.48 years (SD=11.54), with a minimum age of 33 years and a maximum age of 77 years. The average level of education was 7.27 years (SD=4.89), ranging from 0 to 22 years.

Data analysis showed that gender did not influence participant performance. Subject performance was only affected by age on the "Comprehension of Transitive Gestures" subtest (Pearson's correlation p= -0.009), which exhibited a weak but significant correlation.

Education level significantly affected nearly all subtests, as shown in Table 1, where participants with higher education levels generally achieved higher scores.

**Table 1:**
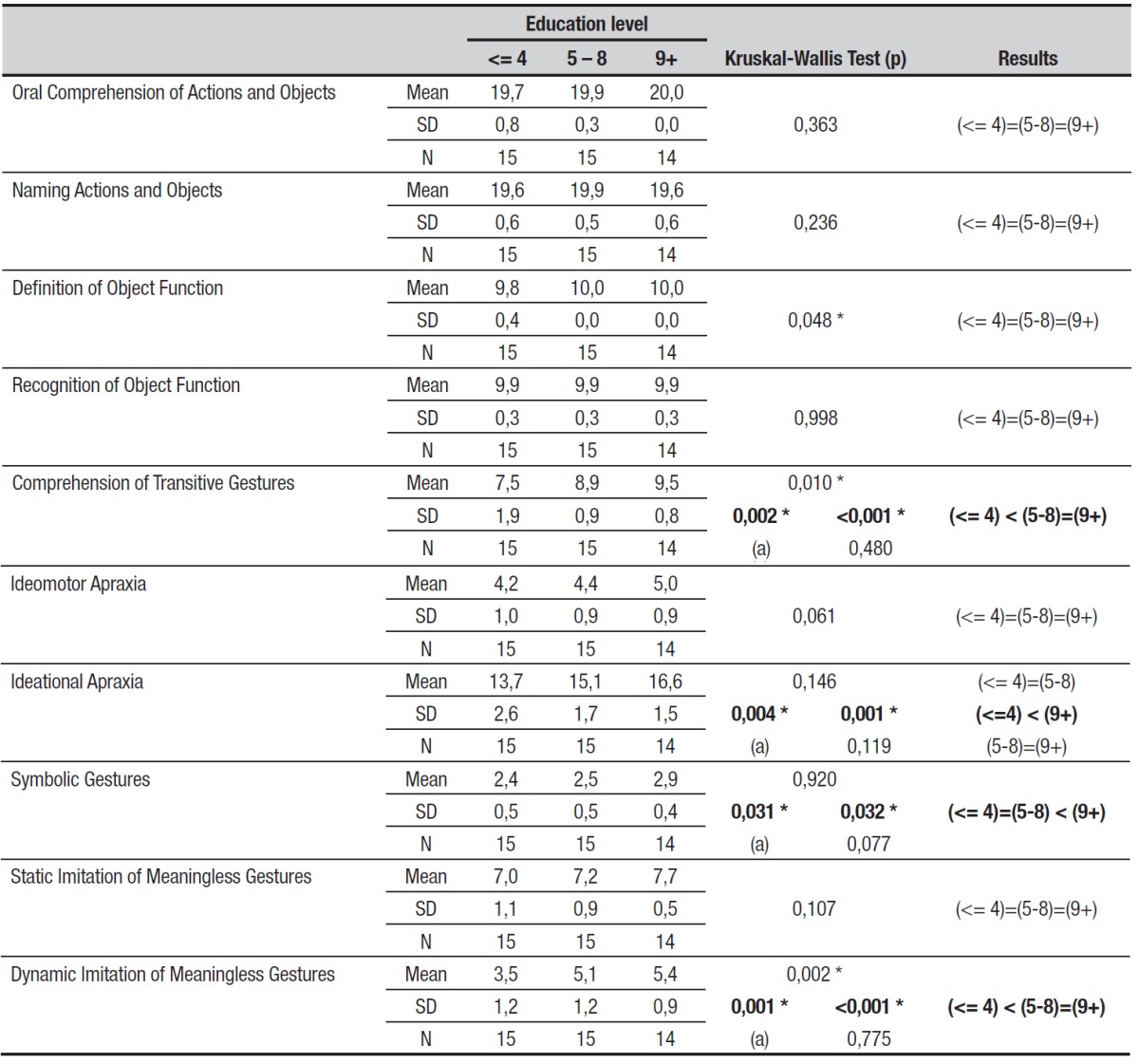
Performance of healthy subjects for each of the subtests in the limb apraxia battery, considering education level and Kruskal-Wallis results.

## DISCUSSION

The strong performance of participants on the subtests that involved strictly linguistic tasks may have been positively affected by the use of photographs, the frequency of objects encountered in daily life, and the fact that certain objects could be handled, all of which facilitate the naming and recognition of objects.^14^ No differences were observed between the performance of men and women for any of the subtests in the battery. With respect to age, only the subtest "Comprehension of Transitive Gestures" was influenced by differences in age. The lower performance by older participants on this subtest could have been due to a number of factors, including deficits in visual processing,^15^ deficits in visual attention, as the photographs showed subtle differences,^16^ or to more general deficits in visuospatial information processing, as there is evidence that this ability decreases with age.^17^ The range of cognitive functions appears to be a continuum that varies over the lifetime of an individual.

With respect to education, correlations were found between performance and education level for all subtests that involved the performance of motor actions. The correlations were weak for the "Symbolic Gestures" and "Static Imitation" subtests, whereas the correlations were moderate for all other subtests.

The "Ideomotor Apraxia" and "Ideational Apraxia" subtests involved the performance of transitive gestures through the use of actual objects or by pantomiming the use of imagined objects, and we observed that the majority of mistakes occurred during the pantomimed performances. The influence of education on the performance of pantomime acts appears to be related to differences in the cognitive skills involved in this task, such as working memory and visuospatial skills, which are likely influenced by low education levels.^16^ ^,^ ^18^ ^,^ ^19^ Furthermore, some authors^20^ claim that the artificiality of the assessment process itself can influence performance, which has been used^17^ as a possible explanation for the poor performance of individuals with low levels of education on neuropsychological tests.

For these actions in the two subtests that involved the use of actual objects, errors occurred less frequently; furthermore, when errors did occur, they were usually caused by unfamiliarity with the object, its function, or its handling, or by failures in the sequence of actions that were directly related to the complexity of the task. In the "Ideational apraxia" subtest, all items included the performing of complex motor actions. In addition to understanding the function and use of an object, a transitive gesture involves both planning the motor acts as well as performing the act itself.^21^ In fact, the existence of a "gestural buffer," or a short-term gestural memory, is assumed, that is related to the time required to translate the abstract idea of a complex gesture into the appropriate sequences of the motor command, which occur in a temporally ordered manner and correspond to the action as a whole.^19^ In addition, deficits in working memory associated with low education levels^17^ or differences in executive functions related to planning and/or attention could also affect performance of these tasks.

We also observed an influence of education on the "Symbolic Gestures" subtest; however, this correlation was weak, which might be due to the small number of items in this test, as the gesture "hail a taxi" was confused several times with the gesture for stopping a bus. Replacing one gesture with another semantically related gesture can indicate a lexical failure.^11^ ^,^ ^19^ However, it is also important to consider the likelihood of exposure to certain lexical gestures, such as the gesture for hailing a taxi, which may not be frequently encountered in the groups with lower levels of education; therefore these gestures might not be engrammed or cognitive access to them might be impaired, causing individuals to use a semantically related gesture that is more common in their daily lives.

Finally, weak and moderate correlations were found between education and the "Static Imitation" and "Dynamic Imitation" subtests, respectively. The production of meaningless gestures involves the processing of gestural information by the non-lexical pathway, which is the visuomotor conversion mechanism by which an observed movement is converted into a motor action without accessing its lexical or semantic components.^19^ During the planning stage of a motor action, the gestural memory buffer is in use, and motor engrams remain active until the gesture is completed. Therefore, to successfully perform an action requires the integration of correctly interpreted visuospatial information and an effective working memory (i.e., the gestural buffer), both of which are cognitive functions that are sensitive to variations in education levels.^15^ ^,^ ^17^ ^,^ ^22^ Similar results were found in a previous study where differences were found during the Luria's Fist Edge-Palm Test according to educational level.^23^

Therefore, we conclude that the education level of participants can have an important influence on the outcome of limb apraxia tests. Gender did not affect the variables tested in this study, and we found some evidence for an age-related decline in performance for one of the tasks in the battery.

## APPENDIX1-LIMBAPRAXIABATTERY

### 1) Lexical-semantic aspects related to gestural production:

1.1. Oral Comprehension of Actions and Objects: out of four photographs shown on a card, the participant must identify the picture corresponding to the action (10 items) or object (10 items) named by the evaluator.

Actions: Comb hair, drink water, cut an onion, eat an apple, cut paper, brush teeth, speak on the phone, light a candle, hammer a nail, ironing.

Objects: Knife, hairbrush, phone, fork, scissors, toothbrush, hammer, matchbox, iron and a cup.

1.2. Naming Actions and Objects: the participant must name the action (10 items) or object (10 items) shown by the evaluator.

Actions: Comb hair, drink water, cut an onion, eat an apple, cut paper, brush teeth, speak on the phone, light a candle, hammer a nail and ironing.

Objects: Knife, hairbrush, phone, fork, scissors, toothbrush, hammer, matchbox, iron and a cup.

1.3. Recognition of Object Function: out of four photographs of objects displayed on a card, the participant must indicate the picture corresponding to the function described by the evaluator.

Objects: Knife, hairbrush, phone, fork, scissors, toothbrush, hammer, matchbox, iron and a cup.

1.4. Definition of Object Function: the participant must describe the function of 10 items shown to them.

Objects: Knife, hairbrush, phone, fork, scissors, toothbrush, hammer, matchbox, iron and a cup.

1.5. Comprehension of Transitive Gestures (i.e., gestures that involve the use of objects): Ten cards, each containing four photographs, are shown to the patient. In each figure, the same person uses the same object; however, in only one of these photos is the object being used correctly in terms of handling and spatial orientation. The participant must recognize and indicate the photo in which the object is being used correctly.

Actions: Comb hair, drink water, cut an onion, eat an apple, cut paper, brush teeth, speak on the phone, light a candle, hammer a nail and ironing.

### 2) Motor activity in response to verbal commands and imitation:^13^

2.1a. Ideomotor apraxia: the participant must demonstrate the use of a given object using gestures. For 3 items, the participant is allowed to hold the object in question in his/her hands. The objects were: paper (to fold), a padlock and a fork. For another 3 items, the evaluator simply states the object and the participant is asked to imagine the object in his/her hands and demonstrate (i.e., pantomime) its use. The objects in this part were a comb, teacup and a toothbrush.

2.1b. Static imitation of meaningless gestures: the participant is asked to imitate the evaluator's hands in three different positions.

b1. Placing hand at a right angle ( ); with palm of the hand facing up ( ); thumbs forming an angle of 90.

b2. Aligning the virtual axis of the thumbs ( ); with fingertips touching each other ( ); and palms of the hands in opposing planes ( ).

b3. Palms of the hands in opposing planes ( ); overlapping hands position ( ).

2.1c. Dynamic imitation of meaningless gestures: the participant is instructed to accurately imitate the hand movements performed by the evaluator.

c1. Palms of hands must be on the same plane ( ); Fingers outstretched in a flying movement ( ).

c2. Fingers form two tweezers ( ); in alternate movements ( ); with his/her hands on a perpendicular plane ( ).

c3. Edge-palm test ( )

2.2. Ideational apraxia: performance of complex gestures is evaluated using 3 objects that are present and 3 objects that are absent (i.e., imagined).

Present: 2.2a. Matchbox: Open the matchbox and take one out ( ); Close the matchbox ( ); Light it ( ).

2.2b. Candle, candle holder, lighter. Grasp the candle in the correct way ( ); place it into the candle holder ( ); Light the candle ( ).

2.2.c. Paper, envelope, stamp. Folding the paper to the envelope size ( ); Open the envelope ( ); Place the paper inside the envelope ( ); Close the envelope ( ); Put the stamp in the correct position.

Absent: 2.2.d. Scissors: hold the paper in one hand ( ); Cut the paper with the other hand ( )

2.2.e. Hammer a nail into the table: holding the nail correctly with one hand ( ); to use the hammer with the other hand ( )

2.2.f. Jug and cup: hold the glass with one hand ( ); hold the jug with another hand ( ); pouring water movement ( ).

2.3. Symbolic gestures: the meaning of three symbolic gestures is assessed: waving hands to say good bye, making a hand gesture to hail a taxi, and gesturing to make someone comes closer.
